# Supplementary material for: Characteristics of the Perianthic Endophytic Fungal Communities of the Rare Horticultural Plant Lirianthe delavayi and Their Changes under Artificial Cultivation
Source: Microorganisms. 2024 Jul 21;12(7):1491. doi: 10.3390/microorganisms12071491 (PMC11278761; doi:10.3390/microorganisms12071491)
Supplement: Supplementary file 1 [file microorganisms-12-01491-s001.zip › microorganisms-3094772-supplementary.pdf]

Characteristics of the Perianthic Endophytic Fungal Communities of the Rare Horticultural Plant *Lirianthe delavayi* and Their Changes under Artificial Cultivation

Lang Yuan [1], Tongxing Zhao [1], Jing Yang [1], Nannan Wu [1], Pinzheng Zhang [1], Hanbo Zhang [2] and Tao Xu [1,]*

1 School of Ecology and Environmental Science, Yunnan University, Kunming 650106, China (yangjing202306@126.com(J.Y.))
2 State Key Laboratory for Conservation and Utilization of Bio-Resources in Yunnan, Yunnan University, Kunming 650106, China
* Correspondence: taoxu@ynu.edu.cn (T.X.)

Supplementary Figures

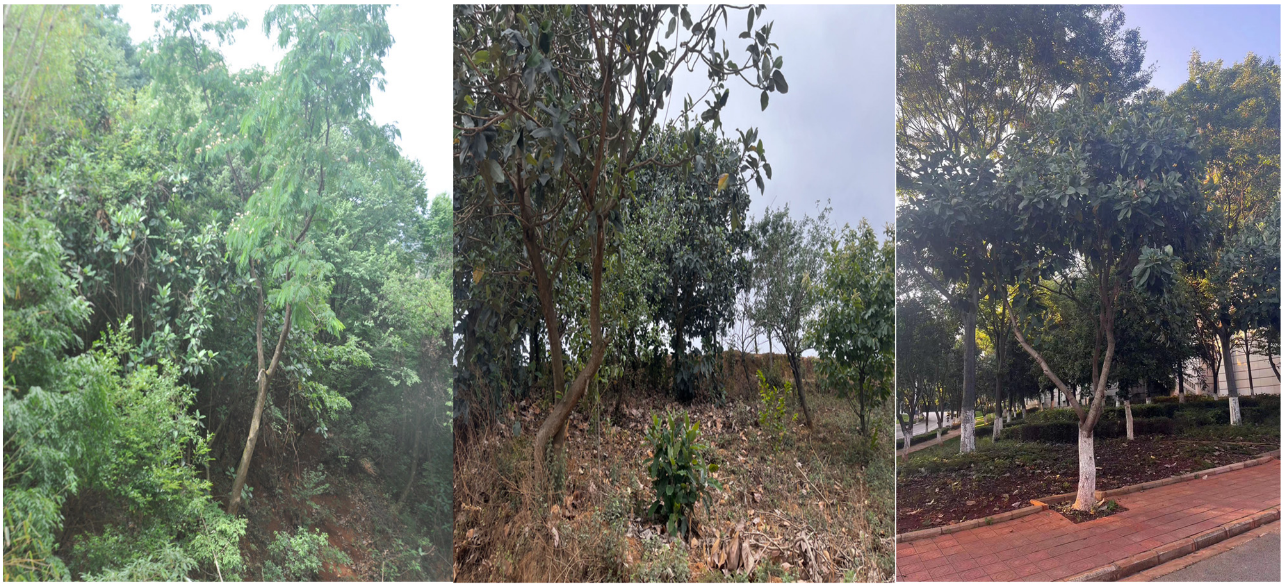

(a) (b) (c)

Figure S1. Habitat of wild *Lirianthe delavayi* in Yunnan Province (a), and cultivated *Lirianthe delavayi* grown in the wild(b), cultivated *Lirianthe delavayi* grown on campus (c).

Supplementary Tables

Table S1. Specific information of *L. delavayi* tepals collected from 31 groups of samples plots in Kunming City, Yunnan Province.

Sample No.	Collection location	N°	E°	Altitude /m	Number of collected samples	Plant species distributed in the surrounding environment
WFU5 (W1)	Adong mountain beside S209 County Road, Majie Town, Yiliang County	25°14′12″	103°11′52″	1804	1	*Ageratina adenophora, Leucaena leucocephala Galinsoga parviflora, Pseudognaphalium affine, Asplenium trichomanes, Pteris cretica* L. var. nervosa, *Artemisia roxburghiana, Oxalis corniculate, Hypoestes trifloral, Hemiphragma heterophyllum, Clematis armandii, Eremochloa ciliaris, Ternstroemia gymnanthera, Hedychium spicatum, Forsythia viridissima, Jasminum grandiflorum, Metapanax delavayi, Hypericum monogynum, Michelia champaca, Dioscorea polystachya*
WFU6 (W2)	Adong mountain beside S209 County Road, Majie Town, Yiliang County	25°14′13″	103°11′50″	1794	1	
WFU7 (W3)	Adong mountain beside S209 County Road, Majie Town, Yiliang County	25°14′13″	103°11′50″	1779	1	
CF1, CFU1 (CX)	Next to Gewu Building, Yunnan University, Chenggong District, Kunming City	24°49′28″	102°51′4″	1975	1	*Celtis kunmingensis* Cheng et Hong, *Euonymus japonicus, Duranta erecta, Loropetalum chinense* var. rubrum, *Rosmarinus officinalis, Ophiopogon japonicus, Cuphea hookeriana, Hedera nepalensis* var. sinensis, *Hydrangea macrophylla, Rose*
CF2, CFU2(CX)	Next to Gewu Building, Yunnan University, Chenggong District, Kunming City	24°49′28″	102°51′3″	1966	1	
CF3, CFU3(CX)	Next to Gewu Building, Yunnan University, Chenggong District, Kunming City	24°49′28″	102°51′3″	1971	1	
CF4, CFU4 (CX)	Next to Gewu Building, Yunnan University, Chenggong District, Kunming City	24°49′27″	102°51′3″	1955	1	
CF5, CFU5(CX)	Next to Gewu Building, Yunnan University, Chenggong District, Kunming City	24°49′27″	102°51′2″	1948	1	
CF6, CFU6 (CX)	Next to Gewu Building, Yunnan University, Chenggong District, Kunming City	24°49′27″	102°51′2″	1945	1	
CF7, CFU7 (CX)	Next to Gewu Building, Yunnan University, Chenggong District, Kunming City	24°49′27″	102°51′2″	1944	1	
CF8, CFU8	Next to Gewu Building,	24°49′27″	102°51′1″	1948	1	

(CX)	Yunnan University, Chenggong District, Kunming City					
CYF1, CYFU1 (CY)	Beside S209 County Road, Majie Town, Yiliang County	25°20'97''	103°19'48''	1815	1	*Zea mays, Artemisia selengensis, Buddleja officinalis Lolium perenne, Sequoia sempervirens, Indocalamus tessellatus, Potentilla discolor.*
CYF2, CYFU2(CY)	Beside S209 County Road, Majie Town, Yiliang County	25°20'97''	103°19'48''	1816	1	
CYF3, CYFU3(CY)	Beside S209 County Road, Majie Town, Yiliang County	25°20'97''	103°19'48''	1815	1	
CYF4, CYFU4 (CY)	Beside S209 County Road, Majie Town, Yiliang County	25°20'97''	103°19'49''	1819	1	
CYF5, CYFU5(CY)	Beside S209 County Road, Majie Town, Yiliang County	25°20'97''	103°19'49''	1820	1	
CYF6, CYFU6 (CY)	Beside S209 County Road, Majie Town, Yiliang County	25°20'97''	103°19'49''	1817	1	
